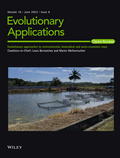# Cover Image

**DOI:** 10.1111/eva.13403

**Published:** 2023-06-22

## Abstract

Caption: Grow‐out ponds for GIFT Nile tilapia, Malaysia.

Credit: Trong Trinh (WorldFish).